# Diagnostic test accuracy of screening tools for the detection of neurocognitive disorders in older adults post-trauma in acute care settings: a systematic review

**DOI:** 10.1007/s41999-025-01287-9

**Published:** 2025-08-18

**Authors:** Niamh A. Merriman, Mary E. Walsh, Helena Ferris, Eithne Sexton, Niamh O’Regan, Rose S. Penfold, Marie Carrigan, Tara Coughlan, Lorna Gurren, Jodie Adams, Chris Reidy, Arveen Jeyaseelan, Patrick Doyle, Mubashra Ashraf, Tomás Ó Flatharta, Siofra Hearne, Jane Gaffey, Louise Brent, Pamela Hickey, Catherine Blake

**Affiliations:** 1 School of Public Health, Physiotherapy and Sports Science, University College Dublin, Dublin, Ireland; 2 School of Pharmacy and Biomolecular Science, Royal College of Surgeons in Ireland University of Medicine and Health Sciences, Dublin, Ireland; 3 Department of Public Health, Health Service Executive - South West, St. Finbarr’s Hospital, Cork, Ireland; 4 School of Population Health, Royal College of Surgeons in Ireland University of Medicine and Health Sciences, Dublin, Ireland; 5 Waterford Integrated Care for Older People, Department of Geriatric Medicine, University Hospital Waterford, Waterford, Ireland; 6 Ageing and Health, Usher Institute, University of Edinburgh and Advanced Care Research Centre, Edinburgh, Scotland UK; 7 Health Information and Quality Authority, Dublin, Ireland; 8 Discipline of Medical Gerontology, School of Medicine, Trinity College Dublin, Dublin, Ireland; 9 Department of Age-Related Healthcare, Tallaght University Hospital, Dublin, Ireland; 10 School of Psychology, Dublin City University, Dublin, Ireland; 11 Department of Population Health Sciences, School of Life Course and Population Sciences, Kings College London, London, UK; 12 Department of Physiotherapy, Guys and St. Thomas’s NHS Foundation Trust, London, UK; 13 National Office of Clinical Audit, Dublin, Ireland

**Keywords:** Systematic review, Diagnostic accuracy, Cognition, Delirium, Hip fracture, Older adults

## Abstract

**Purpose:**

Neurocognitive disorders (NCDs), including delirium, cognitive impairment, or dementia, are prevalent in older patients with physical trauma in acute care settings. Identifying NCDs in these patients can enhance care decisions to improve outcomes. This study aimed to identify the diagnostic accuracy of screening tools for NCDs in older patients with trauma in acute care settings.

**Methods:**

Electronic databases (MEDLINE, Embase, CINAHL, PsycInfo, Cochrane Library) were searched from inception to 01 March 2024. Inclusion criteria were: older adults (≥ 60 years); admitted to acute care setting following physical trauma; diagnostic accuracy study of a screening tool for (1) delirium and/or (2) cognitive impairment or dementia against a reference standard of a clinical diagnosis (standard diagnostic criteria or validated tool). Methodological quality was assessed using the Quality Assessment of Diagnostic Accuracy Studies-2 tool. A narrative synthesis was conducted. Methodological heterogeneity between the studies precluded meta-analysis.

**Results:**

Five studies examining five different screening tools for delirium in older adults, all with hip fracture, were included. Studies reported wide variance in sensitivity (76.9–91.8) and specificity (54.5–99). Prevalence of detected delirium varied widely across studies (6.7–31.5%). All studies had a high or unclear risk of bias in at least one domain. No studies were found to examine the diagnostic accuracy of screening tools for cognitive impairment in older patients with trauma.

**Conclusion:**

This systematic review highlights the dearth of studies validating screening tools for NCDs in older patients following trauma in acute care settings.

**PROSPERO Registration number:**

CRD42024518730.

**Supplementary Information:**

The online version contains supplementary material available at 10.1007/s41999-025-01287-9.

## Key summary points

**Aim:**

To determine the diagnostic accuracy of screening tools for neurocognitive disorders—such as delirium, cognitive impairment, or dementia—in older adults with physical trauma in acute care settings.

**Findings:**

Five studies assessed different screening tools for delirium in older adults with hip fracture. The accuracy of these tools varied widely, with sensitivity ranging from 76.9% to 91.8% and specificity from 54.5% to 99%. The proportion of patients identified with delirium also differed greatly between studies (6.7–31.5%). All studies had a high or unclear risk of bias in at least one area. No studies were found that evaluated screening tools for cognitive impairment or dementia in this cohort.

**Message:**

There is a lack of high-quality research validating screening tools for neurocognitive disorders in older adults after trauma in acute care settings.

**Supplementary Information:**

The online version contains supplementary material available at 10.1007/s41999-025-01287-9.

## Introduction

Trauma is defined as a physical injury of sudden onset and severity which requires immediate medical attention [1]. Over half of patients requiring acute care following physical trauma are over 60 years old [2], with the majority of injuries resulting from low-energy falls of less than 2 m [2]. Delirium is an acute and fluctuating neurocognitive disorder (NCD) characterised by altered levels of awareness, inattention, and other neuropsychiatric disturbances [3]. Dementia is a progressive, major NCD in which there is significant deterioration in cognitive function beyond that which might be expected in biological ageing and which affects independence of function [3, 4]. While cognitive impairment is defined as a mild NCD by Diagnostic and Statistical Manual of Mental Disorders, Fifth Edition (DSM-5) criteria, in which some cognitive function such as memory is disrupted, though functional independence is preserved [3], cognitive impairment is usually referred to in research studies as an impairment in cognitive function identified through positive results on testing that can be due to multiple issues, such as delirium, dementia, or other neuropsychiatric conditions. NCDs that are undetected during an acute hospital admission can lead to poor outcomes, such as increased incidence of depressive symptoms, longer length of stay, and mortality [5, 6]. One study reported a dementia prevalence of 25% in older adults following a hospital admission; however, only 35.6% of patients with dementia had a pre-admission diagnosis [7].

Hip fracture is one of the most common and serious causes of trauma [8], and older adults with hip fracture have complex ongoing healthcare needs, with high prevalences of NCDs [9].

Up to 24% of older adults with hip fracture have a pre-fracture dementia diagnosis, and up to 52% have cognitive impairment (detected by cognitive assessment) [10–13]. There is a substantial number of older adults with hip fracture who have an undiagnosed cognitive impairment, with one study reporting that 63% of older patients with hip fracture screened positive for cognitive impairment, but only 15% of those had a documented diagnosis at the time of hospitalisation [14], indicating probable underdiagnosis in the community.

Older adults with pre-existing dementia or cognitive impairment are at increased risk of developing delirium following physical trauma [15]. Assessment of baseline cognitive function is an essential, though challenging, aspect of clinical care in people with hip fracture and can significantly impact decisions regarding the need for tailored rehabilitation, discharge planning, and the facilitation of focused delirium prevention [16, 17]. As many as 21% of older adults with hip fracture have delirium on admission [18] and 23–51% develop delirium post-operatively [12, 19]. As such, it is vital that pre-operative delirium is not considered an exclusion criterion in diagnostic accuracy studies. Delirium can result in increased length of stay, long-term cognitive and functional decline, increased risk of discharge to long-term care, and higher mortality, in addition to patient and carer distress [20–23]. National clinical guidelines, such as the National Institute for Health and Care Excellence (NICE) and Scottish Intercollegiate Guidelines Network (SIGN), and hip fracture care standards advocate for routine delirium screening with the 4 ‘A’s Test or 4AT (http://www.the4AT.com) [24–27]. Although management of delirium is an essential facet of care for all older adults, those with pre-existing cognitive impairment (either diagnosed or undiagnosed) are at high risk of delirium being undiagnosed [28].

More than 300 delirium or cognitive screening tools currently exist [29, 30]. Several systematic reviews have assessed the diagnostic accuracy of screening tools for delirium [30–32] and cognitive impairment or dementia in older adults in hospital settings [33–35]. A previous review focusing on the diagnostic accuracy of pain and delirium detection tools in older patients with hip fracture and cognitive impairment was published in 2013 [36]. However, that review did not assess for undetected cognitive impairment in an older trauma population. The current systematic review aimed to assess the diagnostic accuracy of screening tools for NCDs in older adults in acute care settings following physical trauma.

## Methods

The systematic review protocol [37] and search strategy were pre-registered with the International Prospective Register of Systematic Reviews (PROSPERO) on 11 March 2024 (https://www.crd.york.ac.uk/prospero/display_record.php?ID=CRD42024518730). The review was carried out and reported in accordance with the Preferred Reporting Items for a Systematic Review and Meta-analysis of Diagnostic Test Accuracy Studies (PRISMA-DTA) checklist [38]. The completed PRISMA-DTA checklist is available in Supplementary Table S1.

### Search strategy and selection criteria

A comprehensive search strategy was developed in collaboration with a librarian, and a search of the following electronic databases was conducted from inception to 01 March 2024: MEDLINE (Ovid), Embase (Elsevier), PsycInfo (EBSCO), CINAHL (EBSCO), and the Cochrane Library (Wiley). The full search strategy is available in Supplementary Table S2. Forward citation searches and hand searches of citation lists of identified systematic reviews and selected included studies were conducted to identify further articles of potential relevance. The eligibility criteria are presented in Table 1.

**Table 1:** Eligibility criteria

*Inclusion criteria*
Patient age ≥ 60 [39]
Main study group or clearly defined subgroup comprising older adults in acute care setting following physical trauma
Examined the diagnostic accuracy of screening tools for detection of (1) delirium or (2) cognitive impairment or dementia
Compared to a reference standard assessment made using standardised diagnostic criteria or a validated tool
Cross-sectional, prospective, or a retrospective cohort study design
Sufficient data provided on the screening tool and reference standard results to construct a two-by-two table of true positives (TP), false positives (FP), true negatives (TN) and false negatives (FN)
*Exclusion criteria*
Focused only on younger people (under 60 years)
Did not include patients with physical trauma
Case reports or case–control design
Non-acute care settings
No full text available

### Study selection

All citations identified from the collective search strategy were exported to EndNote (Version X20) for reference management and de-duplication. Using Covidence Systematic Review Software [40], two reviewers, NAM and another author (RSP, HF, TC, LG, JA, CR, AJ, PD, MA, TOF, SH, JG), independently reviewed the titles and abstracts of the remaining citations to identify those for full-text review. The full texts were obtained and independently evaluated by two reviewers (NAM, MEW) applying the defined inclusion and exclusion criteria. Where disagreements occurred, discussions were held to reach consensus, and where necessary, a third reviewer (CB) was involved. Citations excluded during the full-text review stage were documented alongside the reason for exclusion and included in the PRISMA flow diagram [41].

### Data extraction

Data extraction was performed independently by two reviewers, NAM and another author (MEW, HF). Where disagreements occurred, discussions were held to reach consensus and a third reviewer (CB) was consulted, where necessary. The following data were extracted: (i) title, first author, year and country; (ii) study design; (iii) physical trauma type; (iv) number included in analysis; (v) number of patients with (a) delirium, or (b) number with cognitive impairment or dementia; (vi) patient demographics; (vii) index test (screening tool) and threshold used, if applicable; (viii) reference standard; (ix) index test administration (i.e., timing of index test, healthcare profession responsible for conducting the assessment); (x) reference standard administration; (xi) test accuracy data, extracted in a two-by-two table (TPs, FPs, TNs, FNs; see Supplementary Table S4); (xii) sensitivity and specificity (calculated from two-by-two table).

### Risk of bias assessment

Risk of bias assessment was conducted independently by two reviewers (NAM, ES) using the Quality Assessment of Diagnostic Accuracy Studies (QUADAS-2) tool [42]. This tool comprises four domains: patient selection, index test (screening tool), reference standard, and flow and timing. Each domain is assessed in terms of risk of bias (low, high, or unclear), and the first three domains are also considered in terms of applicability to the current research question. Regarding the flow and timing domain in studies assessing the diagnostic accuracy of delirium screening tools, a 24‐hour period threshold between the reference standard and delirium screening tool administration was pre-specified. Studies reporting an interval of more than 24 h between tests were assessed as at high risk of bias and less than 24 h as low risk. Narrative summaries were generated describing risk of bias (high, low, or unclear) and concerns regarding applicability.

### Data synthesis

Characteristics and results of all studies were narratively synthesised and presented in tables. Statistical analysis was not conducted due to non-comparability of data (i.e., heterogeneity across index tests).

## Results

### Study identification

**Fig. 1:**
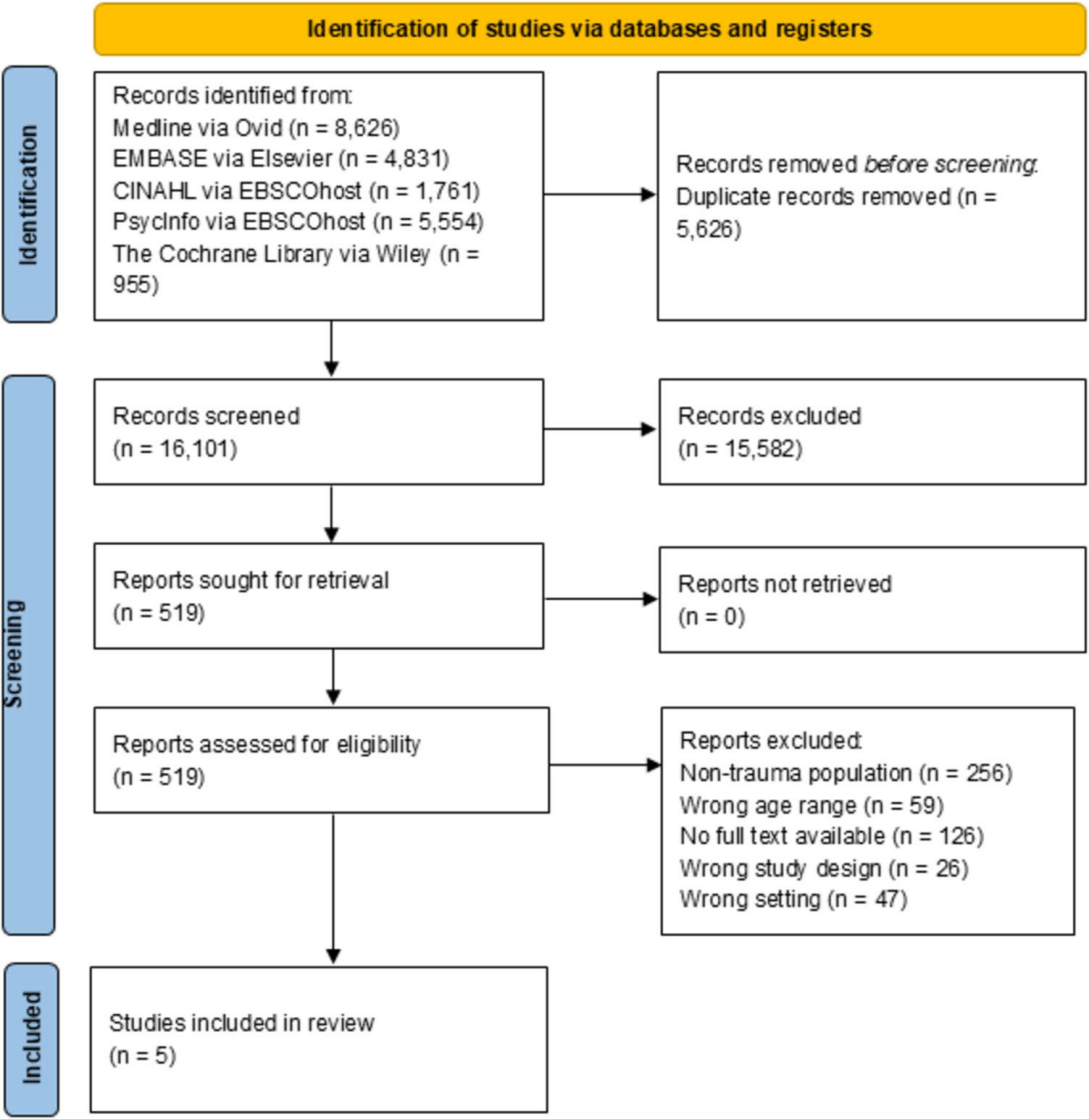
PRISMA flow chart diagram for the search and study selection process

The search of electronic databases from inception to 01 March 2024 identified a total of 21,727 citations. After removal of duplicates, 16,101 records were screened. A total of 519 full text records were assessed for eligibility in accordance with the inclusion and exclusion criteria, from which five studies were identified for inclusion. The main reason for exclusion of articles was that studies did not include a trauma population. The PRISMA flow diagram [43] is presented in Fig. 1.

### Study characteristics

A summary of study characteristics is presented in Table 2. The review cohort comprised 755 participants and 60.6–81.9% were female. Mean or median age of the study population ranged from 77.4 to 86.3 years old. The five included studies all assessed the diagnostic accuracy of screening tools for delirium in older adults with hip fracture in the acute care setting and originated from Belgium [44], Norway [45], the Netherlands [46], Turkey [47], and China [48]. No studies assessing the diagnostic accuracy of assessments for detecting cognitive impairment in older adults following trauma were identified. Various screening tools were investigated, including the Neelon and Champagne (NEECHAM) Confusion Scale [44], the Confusion Assessment Method for the Intensive Care Unit (CAM-ICU) [47], the Delirium Motor Subtype Scale (DMSS) [46], the Mini-Mental State Examination (MMSE) [45], and the Memorial Delirium Assessment Scale (MDAS) [48]. One study used the DSM criteria for delirium as the reference standard [47], while the remaining four studies used the Confusion Assessment Method (CAM) [44–46, 48]. See Supplementary Table S3 for a description of identified screening tools.

**Table 2:** Characteristics of included studies

Author (Country)	Study design	Trauma type	Included in analysis (N)	N (%) delirium	Mean age (SD)	Sex	Index test (Threshold)	Reference standard	Sensitivity specificity (95% CI)	Index test administration	Reference standard administration
Koskderelioglu et al. 2017 (Turkey) [47]	Prospective cohort, consecutive	Hip fracture	109	20 (18.3)	77.4 (7.6)	60.6% female	CAM-ICU (test +)	DSM-IV	80 (56.3–94.3) 98.9 (93.9–100)	From 12 h post-operatively daily until discharge by a neurologist	Administered on the same measurement time points as the index test
Milisen et al. 2005 (Belgium) [44]	Secondary data analysis of control arm of a randomised controlled trial, non-consecutive	Hip fracture	54 (194 obs)	13 obs (6.7)	80.9 (7.9)	81.5% female	NEECHAM (≤ 27)	CAM	76.9 (46.2–95) 64.6 (57.2–71.6)	12 h from admission and on first, third, fifth and eighth post-operative days by trained nursing staff	Administered on the same measurement time points as the index test
Ringdal et al. 2012 (Norway) [45]	Prospective cohort, consecutive	Hip fracture	350	76 (21.7)	84 (median), IQR 65 – 100	75.8% female	MMSE (≤ 23)	CAM	88.4 (78.4–94.9) 54.5 (48.4–60.4)	On same day as CAM in 92% of sample by research team	Daily until sixth post-operative day or discharge
Shi et al. 2014 (China) [48]	Prospective cohort, non-consecutive	Hip fracture	82 (246 obs)	21 (25.6)	80.2 (6.1)	78.0% female	MDAS (≤ 7.5)	CAM	91.8 (80.4–97.3) 99 (96.4–99.9)	First, second, and forth day post-operatively by a psychiatrist	Administered on the same measurement time points as the index test
Slor et al. 2014 (The Netherlands) [46]	Prospective cohort, consecutive	Hip fracture	146	46 (31.5)	86.3 (5.2)*	63.0% female*	DMSS (test +)	CAM	91.3 (79.2–97.6) 87 (78.8–92.9)	Daily from first post-operative day until discharge by member of the research team	Administered on the same measurement time points as the index test

*CAM* Confusion Assessment Method, *CAM-ICU* Confusion Assessment Method for the Intensive Care Unit, *DMSS* Delirium Motor Subtype Scale, *DSM-IV* Diagnostic and Statistical Manual of Mental Disorders IV, *CI* Confidence Intervals, *MDAS* Memorial Delirium Assessment Scale, *MMSE* Mini-Mental Status Examination, *NEECHAM* Neelon and Champagne (NEECHAM) Confusion Scale, *obs* observations, *SD* Standard Deviation

*Figures reported for participants with delirium only

One study administered the index test daily from the first post-operative day until discharge [47], one study screened patients on the first, second, and fourth day post-operatively [48], one study screened patients daily from admission to discharge [46], one study administered the index test on admission and on the first, third, fifth, and eighth post-operative days [44], and one study did not explicitly report the timing of the index test during the acute period [45]. Four studies administered the reference standard on the same measurement time points as the index test [44, 46–48], and one study administered the reference standard daily until the sixth post-operative day or discharge [45]. Two studies reported the combined number of observations of delirium screening across multiple time points when assessing the diagnostic accuracy of the index test [44, 48], while the remaining studies reported one observation per participant in their analyses [45–47]. Index tests were administered by a neurologist [47], trained nursing staff [44], a psychiatrist [48], or the research team [45, 46].

### Diagnostic accuracy

The five included tests for detecting delirium in older adults with trauma in the acute setting demonstrated a wide variance in sensitivity (76.9–91.8) and specificity (54.5–99). Prevalence of detected delirium also varied widely across studies (6.7–31.5%). Extracted test accuracy data are available in Supplementary Table S4. Milisen et al. [44] assessed the Flemish version of the NEECHAM across various thresholds using combined data from five measurement time points. They reported an optimal NEECHAM threshold of 27 or below yielded sensitivity and specificity of 76.9% and 64.6%, respectively. Koskderelioglu et al. [47] examined the Turkish translation of the CAM-ICU and reported sensitivity and specificity as 80% and 98.9%, respectively. Ringdal et al. [45] assessed the Norwegian version of the MMSE and reported sensitivity and specificity of 88.4% and 54.5%, respectively. Slor et al. [46] utilised the Dutch version of the DMSS and reported sensitivity of 91.3% and specificity of 87% on the first post-operative day. Shi et al. [48] examined the diagnostic accuracy of the Chinese translation of the MDAS across various thresholds using combined data across three time points. They reported sensitivity and specificity of 91.8% and 99%, respectively, for an optimal MDAS threshold of 7.5 or below.

### Study quality

**Fig. 2:**
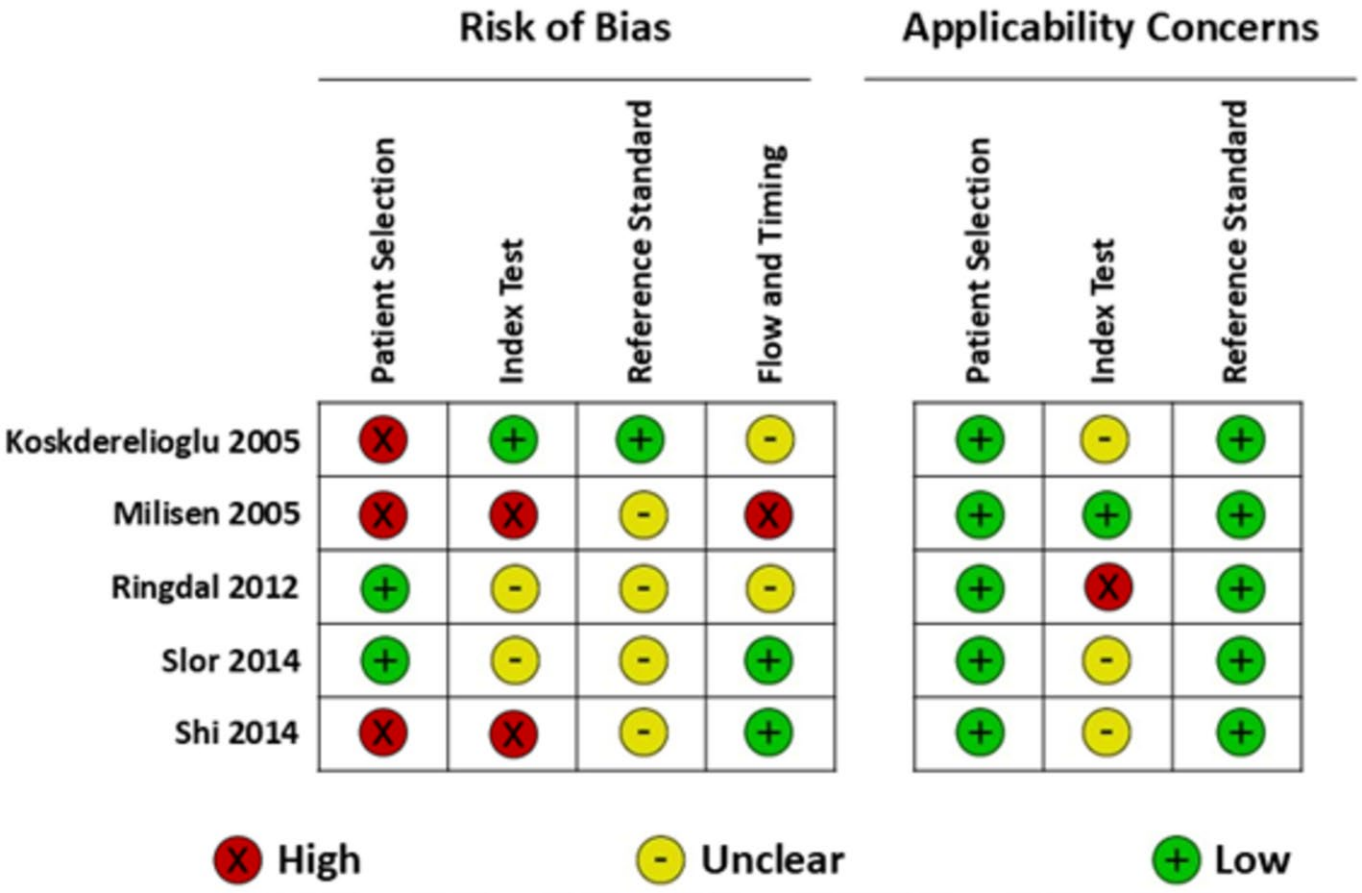
QUADAS-2 risk of bias assessment for included studies (see Supplementary Table S5 for QUADAS-2 assessment criteria)

**Fig. 3:**
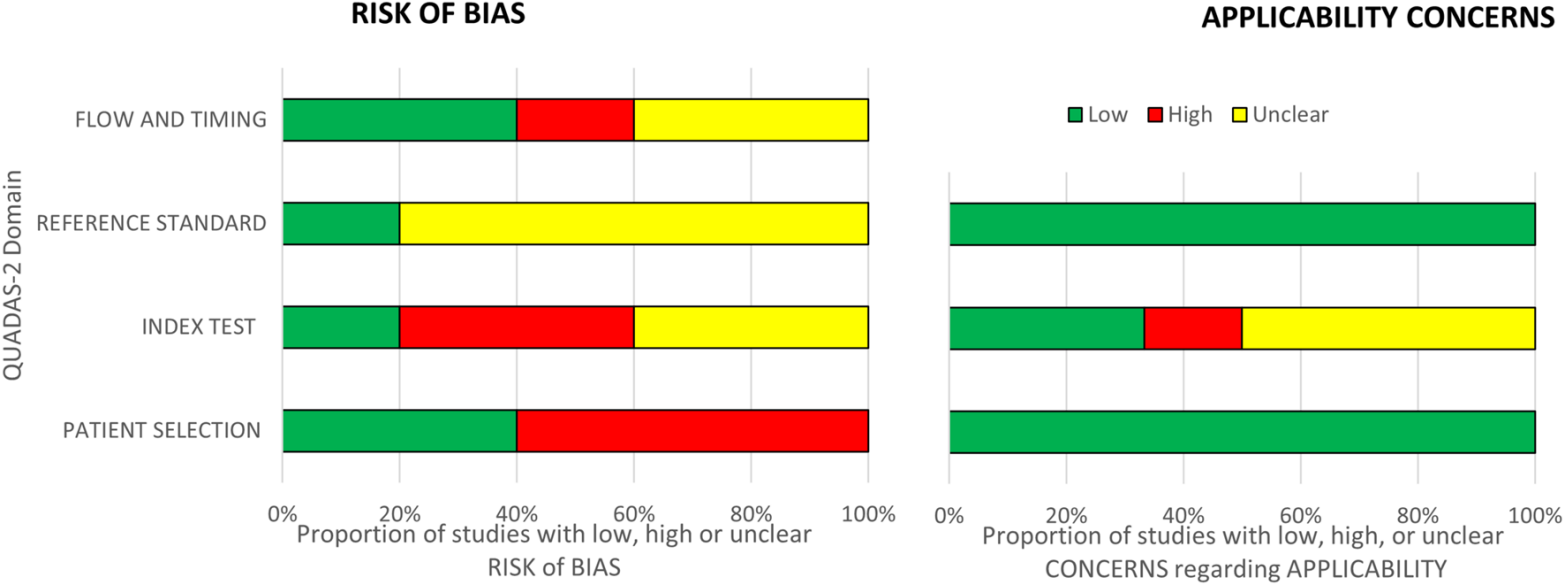
Risk of bias and applicability concerns summary

Results of the risk of bias assessment of the included studies according to the QUADAS-2 criteria are presented in Figs. 2 and 3. All the included studies had a high or unclear assessment of risk of bias in at least one domain. Potential for bias in patient selection included a non-consecutive or non-random sample of patients [44, 48], and the exclusion of patients with delirium on admission and those with cognitive impairment or known dementia [47, 48]. Other areas of concern included insufficient information as to whether the person conducting the index test was blinded to the result of the reference standard [44–46], index test thresholds not being pre-specified [44, 48], insufficient information as to whether the reference standard assessor was blinded to the index test result [44–46, 48], or not all observations included in the analysis [44]. In two studies, the index test and reference standard were administered daily in the post-operative period; however, the authors did not report which measurement timepoint was used of each in the analysis [45, 47]. Two studies repeatedly administered the index test and the combined results were incorporated in the reported sensitivity and specificity [44, 48]. There were applicability concerns regarding the index test in four studies, where the index test was not specifically designed for assessing delirium [45], the index test was not originally designed for assessing delirium outside of critical care settings [47], and where the index test was originally designed for assessing delirium motor subtype or symptom severity [46, 48].

## Discussion

This systematic review aimed to identify, critically appraise, and synthesise the diagnostic accuracy of available screening tools for detection of (1) delirium and (2) cognitive impairment or dementia in adults aged ≥ 60 in acute care settings following physical trauma. A total of five studies comprising 755 participants evaluating the diagnostic accuracy of screening tools for the detection of delirium in older patients with hip fracture across five countries in multiple languages were identified. The prevalence of detected delirium ranged from 6.7% to 31.5% across studies. Reported sensitivity (76.9–91.8) and specificity (54.5–99) of screening tools varied across studies. Given that up to 24% of older adults have a pre-hip fracture diagnosis of dementia [10, 11] and up to 21% have delirium on hospital admission [18], a substantial proportion of the hip fracture population was excluded from two of the five identified studies [47, 48]. Despite the prevalence of cognitive impairment or dementia in older patients with trauma, our findings indicate that cognitive screening instruments have not been evaluated for their diagnostic test accuracy in detecting cognitive impairment in this specific population.

Hip fracture was the trauma in each of the five studies included in this review. This is largely attributable to the fact that hip fracture is one of the most common major traumas in older adults worldwide [8]. Given the prevalence of NCDs in patients with hip fracture and the negative impact on outcomes, it is imperative that screening tools to detect delirium and cognitive impairment are evaluated for their diagnostic accuracy in this population. More specifically, delirium detection continues to be a key health challenge, with underdetection remaining a concern in routine clinical practice [49]. National clinical guidelines (e.g., NICE and SIGN) and hip fracture care standards advocate for routine delirium screening with the 4AT [24–27]. However, this review did not identify any 4AT validation studies specifically in the hip fracture population, although the 4AT has extensive validation data in other settings [31]. Similarly, several hip fracture registries include cognitive assessment data, which is recommended in the Fragility Fracture Network minimum common dataset [12, 50]. However, no validation studies for these assessments were found.

Of the five tools identified for delirium detection, only the CAM-ICU and MMSE are referenced in the SIGN delirium guidelines [25], and of these, only the CAM-ICU is suitable for monitoring delirium. The current review identified one study assessing the diagnostic accuracy of the NEECHAM Confusion Scale. Previous systematic review evidence on the diagnostic potential of the NEECHAM Confusion Scale for detecting delirium is mixed, with one review propounding its use [51], while another recommends against its use due to the methodological flaws of studies assessing its validity [30].

Four of the five identified tools were found to have applicability concerns regarding whether the index test is suitable for detecting delirium in older adults following physical trauma in the acute setting. The CAM-ICU, an adaptation of the CAM, is a tool recommended for use in critical care settings [52]. Although the CAM-ICU has been used to detect delirium in older adults in non-ICU settings, such as by Koskderelioglu et al. [47], further evidence is needed to determine its diagnostic accuracy outside of critical care settings with a physical trauma population [30]. Shi et al. [48] reported the MDAS as having good sensitivity and specificity in detecting delirium in older adults with hip fracture. However, the MDAS was originally designed to assess delirium severity as opposed to delirium detection. Criticism of the MDAS for its use in detecting delirium includes the lack of clarity regarding the optimal cut-off to use as well as the requirement for training and expertise to utilise it, the time taken to conduct the test, and its lack of assessment of the key features of delirium, such as acuity of onset and variability of symptoms, all of which limit its use as a delirium screening tool [51]. The DMSS was also developed to assess delirium severity. While Slor et al. [46] reported the DMSS test accuracy data in detecting delirium, the main aim of the study was to compare the diagnostic accuracy of the DMSS to the Delirium Rating Scale Revised-98 (DRS-R-98) in the identification of delirium motor subtypes in patients diagnosed with delirium. Similarly, the MMSE was developed as a screening tool for cognitive impairment and not for identifying delirium, and so it is unsurprising that the findings of Ringdal et al. [45] suggest that the MMSE does not have delirium diagnostic potential in older adults with hip fracture. Systematic review findings also suggest that the MMSE should not be recommended to detect delirium in hospital settings [53].

While a number of systematic reviews have been conducted on assessment of cognitive function in older adults in acute hospital settings [33–35], this review did not identify any studies that assessed the diagnostic accuracy of screening tools for cognitive impairment in older patients with trauma. Indeed, previous systematic reviews found it difficult to recommend an appropriate cognitive screening tool due to low study quality and heterogeneity of index tests. One in four older adults has a known pre-hip fracture dementia diagnosis, though this is likely an underestimation [7, 14, 54]. Early detection allows for early intervention, discussion with patient and family, community referral for diagnosis, ongoing treatment and post-diagnostic support, and advanced-care planning [35, 55]. However, this should be balanced against valid concerns regarding the potential for misdiagnosis in the acute care setting [35]. Knowledge of pre-fracture cognitive function is essential in hip fracture care and can significantly impact on decisions regarding the need for tailored rehabilitation and discharge planning, as well as facilitating a focused approach to delirium prevention [16, 17]. Furthermore, identifying potential cognitive impairment in the acute care setting serves as an important trigger for referral to memory services or geriatric medicine clinics after discharge, facilitating timely assessment and diagnosis of cognitive impairment or dementia where appropriate [56, 57]. Approximately 80% of national hip fracture registries include a measure of cognitive function as part of routine data collection, demonstrating that assessment in the acute phase is both practical and widely implemented [12].

This review used a comprehensive search strategy to identify relevant studies. Additionally, case–control studies were excluded due to the risk of overestimation of the diagnostic accuracy of the index test. The methodological rigour of the review process was enhanced through independent citation screening, full text review, data extraction, and risk of bias assessment. A key finding of the review is the heterogeneity of identified screening tools. However, due to non-comparable data relating to index tests, it was not appropriate to pool the data in a meta-analysis. It is important to acknowledge the limitations in our study. The requirement for a robust reference standard, coupled with delirium’s fluctuating nature necessitating frequent assessments, limited inclusion of well-cited delirium studies as well as those studies whose focus was on feasibility and applicability. Notably, some widely used delirium screening tools, such as the 4AT, were not included in our review. This omission underscores the need for further investigation of these tools, particularly in older patients with trauma. The absence of such commonly employed instruments from our analysis may limit the immediate clinical applicability of our findings.

The current review identified only five validated instruments for detecting delirium and none for detecting cognitive impairment in older patients with trauma. Existing evidence is therefore insufficient to recommend appropriate screening tools for NCDs in this population. There is a clear need for good quality validation studies of NCD screening tools for older patients with trauma in the acute care setting, particularly when assessing cognitive function. The 4AT has demonstrated substantial validity as a delirium assessment instrument in older adults and is widely accepted as one of the best tools to use in clinical practice due to its brevity and ease of use [31, 49, 58]. Approximately 32% of national hip fracture registries have implemented the 4AT in routine clinical practice [12, 59, 60], with the National Hip Fracture Database now collecting the results of all four domains of the 4AT separately, using the 4-item ‘AMT4’ [61] sub-domain of the 4AT as an indicator of cognitive impairment. A recent large cohort study of hospitalised older adults showed that 4AT scores in both the cognitive impairment (scores 1–3) and delirium (scores ≥ 4) ranges were strongly associated with clinically diagnosed dementia, supporting the use of routine 4AT screening to help identify older adults who may benefit from further cognitive assessment or specialist follow-up [62]. Future research could collect diagnostic accuracy data on the 4AT during routine hip fracture care to further support its use in this cohort.

## Conclusion

This systematic review highlights the dearth of studies validating screening tools for neurocognitive disorders in older adults following physical trauma in acute care settings, despite their widespread use in routine hip fracture care. Due to the limited diagnostic accuracy for each delirium screening tool and unclear or high risk of bias in the included studies, as well as the lack of studies validating screening tools for cognitive impairment in older trauma patients, more evidence is needed to determine the appropriate NCD screening tools for use in this population.

## Supplementary Information

Below is the link to the electronic supplementary material.

Supplementary file1 (DOCX 51 KB)

## Data Availability

Data available within the article or its supplementary materials.
