# Supplementary material for: Diagnostic test accuracy of screening tools for the detection of neurocognitive disorders in older adults post-trauma in acute care settings: a systematic review
Source: Eur Geriatr Med. 2025 Aug 18;17(2):525–35. doi: 10.1007/s41999-025-01287-9 (PMC13109106; doi:10.1007/s41999-025-01287-9)
Supplement: Supplementary file 1 — Supplementary file1 (DOCX 51 KB) [file 41999_2025_1287_MOESM1_ESM.docx]

**Diagnostic test accuracy of screening tools for the detection of neurocognitive disorders in older adults post-trauma in acute care settings: A systematic review**

**European Geriatric Medicine**

**Author names:**

Niamh A. Merriman^1^

Mary E. Walsh^1,2^

Helena Ferris^3^

Eithne Sexton^4^

Niamh O’Regan^5^

Rose S. Penfold^6^

Marie Carrigan^7^

Tara Coughlan^8,9^

Lorna Gurren^10^

Jodie Adams^11,12^

Chris Reidy^5^

Arveen Jeyaseelan^5^

Patrick Doyle^5^

Mubashra Ashraf^5^

Tomás Ó Flatharta^5^

Siofra Hearne^5^

Jane Gaffey^10^

Louise Brent^4,13^

Pamela Hickey^13^

Catherine Blake^1^

**Affiliations:**

^1^School of Public Health, Physiotherapy and Sports Science, University College Dublin, Dublin, Ireland

^2^School of Pharmacy and Biomolecular Science, Royal College of Surgeons in Ireland University of Medicine and Health Sciences, Dublin, Ireland

^3^Department of Public Health, Health Service Executive - South West, St. Finbarr's Hospital, Cork, Ireland

^4^School of Population Health, Royal College of Surgeons in Ireland University of Medicine and Health Sciences, Dublin, Ireland

^5^Waterford Integrated Care for Older People, Department of Geriatric Medicine, University Hospital Waterford, Waterford, Ireland

^6^Ageing and Health, Usher Institute, University of Edinburgh and Advanced Care Research Centre, Edinburgh, Scotland, United Kingdom

^7^Health Information and Quality Authority, Dublin, Ireland

^8^Discipline of Medical Gerontology, School of Medicine, Trinity College Dublin, Dublin, Ireland

^9^Department of Age-related Healthcare, Tallaght University Hospital, Dublin, Ireland

^10^School of Psychology, Dublin City University, Dublin, Ireland

^11^Department of Population Health Sciences, School of Life Course and Population Sciences, Kings College London, London, United Kingdom

^12^Department of Physiotherapy, Guys and St Thomas’s NHS Foundation Trust, London, United Kingdom

^13^National Office of Clinical Audit, Dublin, Ireland

**Corresponding author:**

Dr Niamh A. Merriman

School of Public Health, Physiotherapy and Sports Science,

University College Dublin,

Dublin 4,

Ireland

Email: [niamh.merriman1@ucd.ie](mailto:niamh.merriman1@ucd.ie)

**SUPPLEMENTARY INFORMATION**

**Supplementary Table S1**: Preferred Reporting Items for a Systematic Review and Meta-analysis of Diagnostic Test Accuracy Studies (PRISMA-DTA) checklist

**Supplementary Table S2**: Search Strategy

**Supplementary Table S3**: Description of identified delirium screening tools

**Supplementary Table S4**: Test accuracy data

**Supplementary Table S5**: QUADAS-2 assessment criteria

**SUPPLEMENTARY TABLE** S1: Preferred Reporting Items for a Systematic Review and Meta-analysis of Diagnostic Test Accuracy Studies (PRISMA-DTA) checklist.

| **Section/topic** | **#** | **PRISMA-DTA Checklist Item** | **Reported on page #** |
| --- | --- | --- | --- |
| **TITLE / ABSTRACT** | | |  |
| Title | 1 | Identify the report as a systematic review (+/- meta-analysis) of diagnostic test accuracy (DTA) studies. | 1 |
| Abstract | 2 | Abstract: See PRISMA-DTA for abstracts. | 3 |
| **INTRODUCTION** | | |  |
| Rationale | 3 | Describe the rationale for the review in the context of what is already known. | 4-5 |
| Clinical role of index test | D1 | State the scientific and clinical background, including the intended use and clinical role of the index test, and if applicable, the rationale for minimally acceptable test accuracy (or minimum difference in accuracy for comparative design). | 4-5 |
| Objectives | 4 | Provide an explicit statement of question(s) being addressed in terms of participants, index test(s), and target condition(s). | 6 |
| **METHODS** | | |  |
| Protocol and registration | 5 | Indicate if a review protocol exists, if and where it can be accessed (e.g., Web address), and, if available, provide registration information including registration number. | 6 |
| Eligibility criteria | 6 | Specify study characteristics (participants, setting, index test(s), reference standard(s), target condition(s), and study design) and report characteristics (e.g., years considered, language, publication status) used as criteria for eligibility, giving rationale. | 7 |
| Information sources | 7 | Describe all information sources (e.g., databases with dates of coverage, contact with study authors to identify additional studies) in the search and date last searched. | 7 |
| Search | 8 | Present full search strategies for all electronic databases and other sources searched, including any limits used, such that they could be repeated. | 7 |
| Study selection | 9 | State the process for selecting studies (i.e., screening, eligibility, included in systematic review, and, if applicable, included in the meta-analysis). | 7-8 |
| Data collection process | 10 | Describe method of data extraction from reports (e.g., piloted forms, independently, in duplicate) and any processes for obtaining and confirming data from investigators. | 8 |
| Definitions for data extraction | 11 | Provide definitions used in data extraction and classifications of target condition(s), index test(s), reference standard(s) and other characteristics (e.g. study design, clinical setting). | 8 |
| Risk of bias and applicability | 12 | Describe methods used for assessing risk of bias in individual studies and concerns regarding the applicability to the review question. | 8 |
| Diagnostic accuracy measures | 13 | State the principal diagnostic accuracy measure(s) reported (e.g. sensitivity, specificity) and state the unit of assessment (e.g. per-patient, per-lesion). | 8 |
| Synthesis of results | 14 | Describe methods of handling data, combining results of studies and describing variability between studies. This could include, but is not limited to: a) handling of multiple definitions of target condition. b) handling of multiple thresholds of test positivity, c) handling multiple index test readers, d) handling of indeterminate test results, e) grouping and comparing tests, f) handling of different reference standards | 8 |
| Meta-analysis | D2 | Report the statistical methods used for meta-analyses, if performed. | NA |
| Additional analyses | 16 | Describe methods of additional analyses (e.g., sensitivity or subgroup analyses, meta-regression), if done, indicating which were pre-specified. | NA |
| **RESULTS** | | |  |
| Study selection | 17 | Provide numbers of studies screened, assessed for eligibility, included in the review (and included in meta-analysis, if applicable) with reasons for exclusions at each stage, ideally with a flow diagram. | 9 |
| Study characteristics | 18 | For each included study provide citations and present key characteristics including: a) participant characteristics (presentation, prior testing), b) clinical setting, c) study design, d) target condition definition, e) index test, f) reference standard, g) sample size, h) funding sources | 10 |
| Risk of bias and applicability | 19 | Present evaluation of risk of bias and concerns regarding applicability for each study. | 14-15 |
| Results of individual studies | 20 | For each analysis in each study (e.g. unique combination of index test, reference standard, and positivity threshold) report 2x2 data (TP, FP, FN, TN) with estimates of diagnostic accuracy and confidence intervals, ideally with a forest or receiver operator characteristic (ROC) plot. | 14 |
| Synthesis of results | 21 | Describe test accuracy, including variability; if meta-analysis was done, include results and confidence intervals. | 14 |
| Additional analysis | 23 | Give results of additional analyses, if done (e.g., sensitivity or subgroup analyses, meta-regression; analysis of index test: failure rates, proportion of inconclusive results, adverse events). | NA |
| **DISCUSSION** | | |  |
| Summary of evidence | 24 | Summarize the main findings including the strength of evidence. | 17 |
| Limitations | 25 | Discuss limitations from included studies (e.g. risk of bias and concerns regarding applicability) and from the review process (e.g. incomplete retrieval of identified research). | 19 |
| Conclusions | 26 | Provide a general interpretation of the results in the context of other evidence. Discuss implications for future research and clinical practice (e.g. the intended use and clinical role of the index test). | 17-19 |
| **FUNDING** | | |  |
| Funding | 27 | For the systematic review, describe the sources of funding and other support and the role of the funders. | 20 |

**SUPPLEMENTARY TABLE** S2: Search Strategy.

Resources: Ovid MEDLINE(R) Epub Ahead of Print and Ovid MEDLINE(R) 1946 to 11 March 2024.

| 1 | ("4AT" or "4 AT" or "4AST" or "4 AS" or "4 A S test").ti,ab. |
| --- | --- |
| 2 | "confusion assessment method".ti,ab. |
| 3 | ("CAM" or "CAMICU" or "3DCAM" or "BCAM" or "UBCAM" or "UB2CAM").ti,ab. |
| 4 | ("CAM-ICU" or "3D-CAM" or "B-CAM" or "UB-CAM" or "UB2-CAM").ti,ab. |
| 5 | ("delirium observation screening scale" or "DOSS").ti,ab. |
| 6 | ("Single Question" or "SQID" or "squid").ti,ab. |
| 7 | ("recognizing acute delirium" or "recognising acute delirium" or "RADAR").ti,ab. |
| 8 | ("intensive care delirium screening checklist" or "ICDSC" or "ICD-SC").ti,ab. |
| 9 | "nursing delirium screening scale".ti,ab. |
| 10 | ("NUDESC" or "NU-DESC" or "NDESC" or "N DESC").ti,ab. |
| 11 | or/1-10 |
| 12 | ((screen* or assess* or detect* or diagnos*) adj3 (scale* or score* or tool* or test*)).ab,ti. |
| 13 | exp Mass Screening/ |
| 14 | Early Diagnosis/ |
| 15 | or/12-14 |
| 16 | 11 or 15 |
| 17 | exp Delirium/ |
| 18 | deliri*.ti,ab. |
| 19 | (confus* adj3 state*).ti,ab. |
| 20 | (acute* adj3 (confus* or "brain syndrome" or "brain failure")).ti,ab. |
| 21 | organic psychosyndrome.ti,ab. |
| 22 | psychoorganic syndrome.ti,ab. |
| 23 | psycho-organic syndrome.ti,ab. |
| 24 | toxic confusion*.ti,ab. |
| 25 | toxic psychosis.ti,ab. |
| 26 | or/17-25 |
| 27 | exp "Sensitivity and Specificity"/ |
| 28 | sensitivity.tw. |
| 29 | specificity.tw. |
| 30 | ((pre-test or pretest) adj probability).tw. |
| 31 | post-test probability.tw. |
| 32 | predictive value*.tw. |
| 33 | likelihood ratio*.tw. |
| 34 | or/27-33 |
| 35 | 16 and 26 and 34 |
| 36 | "Addenbrooke* Cognitive Exam*".ti,ab. |
| 37 | "ACE-R".ti,ab. |
| 38 | "mini-ACE".ti,ab. |
| 39 | "ACE-III".ti,ab. |
| 40 | "word recall".ti,ab. |
| 41 | ("10 point cognitive screener" or "10-CS").ti,ab. |
| 42 | "7‐minute screen".ti,ab. |
| 43 | "6 item cognitive impairment test".ti,ab. |
| 44 | "6 CIT".ti,ab. |
| 45 | "cognitive screen".ti,ab. |
| 46 | "abbreviated mental test".ti,ab. |
| 47 | "AMT".ti,ab. |
| 48 | "AMTS".ti,ab. |
| 49 | "ADAS‐cog".ti,ab. |
| 50 | AD8.ti,ab. |
| 51 | "inform* interview".ti,ab. |
| 52 | "animal fluency test".ti,ab. |
| 53 | "brief alzheimer* screen".ti,ab. |
| 54 | "brief cognitive scale".ti,ab. |
| 55 | "clinical dementia rating scale".ti,ab. |
| 56 | "clinical dementia test".ti,ab. |
| 57 | "cognitive abilities screening instrument".ti,ab. |
| 58 | "cognitive assessment screening test".ti,ab. |
| 59 | "cognitive capacity screening examination".ti,ab. |
| 60 | "clock drawing test".ti,ab. |
| 61 | "deterioration cognitive observee".ti,ab. |
| 62 | "Dem Tect".ti,ab. |
| 63 | "fuld object memory evaluation".ti,ab. |
| 64 | "IQCODE".ti,ab. |
| 65 | "mattis dementia rating scale".ti,ab. |
| 66 | "memory impairment screen".ti,ab. |
| 67 | "minnesota cognitive acuity screen".ti,ab. |
| 68 | "mini‐cog".ti,ab. |
| 69 | "mini‐mental state exam*".ti,ab. |
| 70 | ("mmse" or "smmse").ti,ab. |
| 71 | ("modified mini‐mental state exam*" or "standardised mini‐mental state exam*" or "standardized mini‐mental state exam*").ti,ab. |
| 72 | "montreal cognitive assessment".ti,ab. |
| 73 | "moca".ti,ab. |
| 74 | "3MS".ti,ab. |
| 75 | "neurobehavioural cognitive status exam*".ti,ab. |
| 76 | "cognistat".ti,ab. |
| 77 | "quick cognitive screening test".ti,ab. |
| 78 | "QCST".ti,ab. |
| 79 | "rapid dementia screening test".ti,ab. |
| 80 | "RDST".ti,ab. |
| 81 | "repeatable battery for the assessment of neuropsychological status".ti,ab. |
| 82 | "RBANS".ti,ab. |
| 83 | "rowland universal dementia assessment scale".ti,ab. |
| 84 | "rudas".ti,ab. |
| 85 | "self‐administered gerocognitive exam*".ti,ab. |
| 86 | ("self‐administered" and "SAGE").ti,ab. |
| 87 | "short and sweet screening instrument".ti,ab. |
| 88 | "sassi".ti,ab. |
| 89 | "short cognitive performance test".ti,ab. |
| 90 | "syndrome kurztest".ti,ab. |
| 91 | "six item screener".ti,ab. |
| 92 | "short memory questionnaire".ti,ab. |
| 93 | "short orientation memory concentration test".ti,ab. |
| 94 | "s‐omc".ti,ab. |
| 95 | "short blessed test".ti,ab. |
| 96 | "short portable mental status questionnaire".ti,ab. |
| 97 | "spmsq".ti,ab. |
| 98 | "short test of mental status".ti,ab. |
| 99 | ("test your memory" or "TYM").ti,ab. |
| 100 | "trail making test".ti,ab. |
| 101 | "verbal fluency categories".ti,ab. |
| 102 | "WORLD test".ti,ab. |
| 103 | "Hopkins verbal learning test".ti,ab. |
| 104 | "HVLT".ti,ab. |
| 105 | "time and change test".ti,ab. |
| 106 | "modified world test".ti,ab. |
| 107 | "symptoms of dementia screener".ti,ab. |
| 108 | "dementia questionnaire".ti,ab. |
| 109 | "7MS".ti,ab. |
| 110 | ("concord informant dementia scale" or CIDS).ti,ab. |
| 111 | (SAPH or "dementia screening and perceived harm*").ti,ab. |
| 112 | "Ottawa 3DY".ab,ti. |
| 113 | "O3DY".ab,ti. |
| 114 | or/36-113 |
| 115 | 15 or 114 |
| 116 | exp Dementia/ |
| 117 | exp Cognitive Dysfunction/ or exp Memory Disorders/ |
| 118 | dementi*.ti,ab. |
| 119 | alzheimer*.ti,ab. |
| 120 | ("lewy bod*" or DLB or LBD).ti,ab. |
| 121 | ((cognit* or memory or cerebr* or mental*) adj3 (impair* or declin* or function* or los* or deteriorate* or degenerate* or complain* or disturb* or disorder*)).ti,ab. |
| 122 | or/116-121 |
| 123 | 34 and 115 and 122 |
| 124 | 35 or 123 |

**SUPPLEMENTARY TABLE** S3: Description of identified delirium screening tools.

| **Instruments** | **NEECHAM [1]** | **MMSE [2]** | **CAM-ICU [3]** | **MDAS [4]** | **DMSS [5]** |
| --- | --- | --- | --- | --- | --- |
| **Target population** | Older adults in a hospital setting. | Adults in a hospital setting, community setting. | Adults in an ICU setting. | Adults in a hospital setting. | Adults in a hospital setting. |
| **Features** | - Processing: attention, command, orientation. - Behaviour: appearance, motor, verbal. - Physiological control: vital functions, oxygen saturation, continence | - Attention and orientation - Memory - Registration - Recall - Calculation - Language - Visual construction | - Acute change in mental status or ﬂuctuation in the level of consciousness over the prior 24h - Inattention - Disorganized thinking - Altered level of consciousness | - Awareness - Orientation - Short-term memory - Digit span - Attention - Organisational thinking - Perceptual disturbance - Delusions - Sleep-wake cycle - Psychomotor activity | - Hyperactive subtype: increased motor activity, loss of control of activity, restlessness, wandering - Hypoactive subtype: decreased activity, decreased speed of actions, reduced awareness, decreased speech, slowed speech, listlessness, reduced alertness - Mixed motor subtype - No motor subtype |
| **Scoring systems/**  **cutoff point** | - Range: 0-30 - Each item is rated from 0-2, 0-4, or 0-5. - 27-30: no delirium - 25-26: at risk - 20-24: early to mild delirium - A total score ≤19 indicates moderate to severe delirium. | - Range: 0-30 - Each item is rated from 0–1 - 24-30: no cognitive impairment - 18-23: mild cognitive impairment - 0-17: severe cognitive impairment | - Positive or negative for delirium - Each item is rated yes or no - Positive for delirium if Features 1 and 2 and either Feature 3 or Feature 4 are identiﬁed | - Range: 0-30 - Each item is rated from 0-3. - Scores ≥13 indicate the presence of delirium | - Each item is rated as present or absent - Positive for hyperactive delirium if two or more hyperactive features present - Positive for hypoactive delirium if two or more hypoactive features present. At least one of either decreased activity or decreased speed must be present - Mixed motor subtype present if evidence of both hyper- and hypoactive delirium - No motor subtype if no evidence of hyper- or hypoactive delirium |
| **Time need** | 8-10 minutes | 5-10 minutes | <2 minutes | 10-15 minutes | 20‐30 minutes |
| **Assessment approach** | Based on nursing staff observation and interaction within a 24-hour period. | Based on the observation at one time-point. | Based on the observation at one time-point.  (Need baseline cognitive function and additional information from examinations carried out routinely in critical care assessments, such as the Richmond Agitation-Sedation Scale (RASS)) | Based on the observation at one time-point | Based on the observation at one time-point to evaluate the preceding 24-hour period |

**SUPPLEMENTARY TABLE** S4: Test accuracy of included studies.

| **Author (Country)** | **Index Test (Threshold)** | **Reference Standard** | **Included in Analysis (N)** | **TP** | **FN** | **TN** | **FP** | **N (%) delirium** | **Sensitivity**  **Specificity** |
| --- | --- | --- | --- | --- | --- | --- | --- | --- | --- |
| Koskderelioglu et al. 2017 (Turkey) [6] | CAM-ICU  (test +) | DSM-IV | 109 | 16 | 4 | 1 | 88 | 20 (18.3) | 80 (56.3 – 94.3)  98.9 (93.9 – 100) |
| Milisen et al. 2005 (Belgium) [7] | NEECHAM  (≤ 27) | CAM | 54 (194 obs) | 10 | 3 | 64 | 117 | 13 obs  (6.7) | 76.9 (46.2 – 95)  64.6 (57.2 – 71.6) |
| Ringdal et al. 2012 (Norway) [8] | MMSE  (≤23) | CAM | 350 | 61 | 8 | 128 | 153 | 76 (21.7) | 88.4 (78.4 – 94.9)  54.5 (48.4 – 60.4) |
| Shi et al. 2014 (China) [9] | MDAS  (≤ 7.5) | CAM | 82 (246 obs) | 45 | 2 | 4 | 195 | 21 (25.6) | 91.8 (80.4 – 97.3)  99 (96.4 – 99.9) |
| Slor et al. 2014 (The Netherlands) [10] | DMSS  (test +) | CAM | 146 | 42 | 4 | 13 | 87 | 46 (31.5) | 91.3 (79.2 – 97.6)  87 (78.8 – 92.9) |

**Abbreviations**: obs – observations; FN – False Negatives; FP – False Positives; TN – True Negatives; TP – True Positives

**SUPPLEMENTARY TABLE** S5: QUADAS-2 assessment criteria.

| **Domain** | **Patient selection** | **Index test** | **Reference standard** | **Flow and timing** |
| --- | --- | --- | --- | --- |
| Description | Describe methods of patient selection. | Describe the index test and how it was conducted and interpreted. | Describe the reference standard and how it was conducted and interpreted. | Describe any patients who  did not receive the index  test(s) and/or reference  standard or who were excluded from the 2 x 2 table (refer to flow diagram). |
| Signalling questions (yes, no, unclear) | Was a consecutive or random sample of patients enrolled?  Was a case-control design  avoided?  Did the study avoid inappropriate exclusions? | Were the index test results interpreted without knowledge of the results of the reference standard?  If a threshold was used, was it pre-specified? | Is the reference standard likely to correctly classify the target condition?  Were the reference standard results interpreted without  knowledge of the results of the index test? | Was there an appropriate interval between index test(s) and reference standard (i.e., <24 hours between delirium screening tool administration and reference standard)?  Did all patients receive the same reference standard?  Were all patients included in the analysis? |
| Risk of bias | Could the selection of patients have introduced  bias? | Could the conduct or interpretation of the index  test have introduced  bias? | Could the reference standard, its conduct, or its interpretation have introduced  bias? | Could the patient flow have introduced bias? |
| Concerns regarding applicability | Are there concerns that  the included patients do not match the review question? | Are there concerns that  the index test, its conduct,  or its interpretation differ from the review question? | Are there concerns that the target condition as defined by the reference standard does not match the review question? | — |

**References**

1. Neelon VJ, Champagne MT, Carlson JR, Funk SG. The NEECHAM Confusion Scale: Construction, Validation, And Clinical Testing. Nurs Res. 1996;45(6).

2. Folstein M, Folstein S, McHugh P. “Mini-mental state”: a practical method for grading the cognitive state of patients for the clinician. J Psychiatr Res. 1975;12(3):189-98.

3. Ely EW, Margolin R, Francis J, May L, Truman B, Dittus R, et al. Evaluation of delirium in critically ill patients: Validation of the Confusion Assessment Method for the Intensive Care Unit (CAM-ICU). Crit Care Med. 2001;29(7).

4. Breitbart W, Rosenfeld B, Roth A, Smith MJ, Cohen K, Passik S. The memorial delirium assessment scale. J Pain Symptom Manage. 1997;13(3):128-37. <https://doi.org/10.1016/S0885-3924(96)00316-8>

5. Meagher D, Moran M, Raju B, Gibbons D, Donnelly S, Saunders J, et al. A new data-based motor subtype schema for delirium. J Neuropsychiatry Clin Neurosci. 2008;20:185-93.

6. Koskderelioglu A, Onder O, Gucuyener M, Altay T, Kayali C, Gedizlioglu M. Screening for postoperative delirium in patients with acute hip fracture: Assessment of predictive factors. Geriatr Gerontol Int. 2017;17(6):919-24. <https://doi.org/10.1111/ggi.12806>

7. Milisen K, Foreman MD, Hendrickx A, Godderis J, Abraham IL, Broos PLO, et al. Psychometric properties of the Flemish translation of the NEECHAM Confusion Scale. BMC Psychiatry. 2005;5(1):16. <http://doi.org/10.1186/1471-244X-5-16>

8. Ringdal GI, Ringdal K, Juliebø V, Wyller TB, Hjermstad MJ, Loge JH. Using the Mini-Mental State Examination to Screen for Delirium in Elderly Patients with Hip Fracture. Dement Geriatr Cogn Disord. 2012;32(6):394-400. <http://doi.org/10.1159/000335743>

9. Shi Z, Wu Y, Li C, Fu S, Li G, Zhu Y, et al. Using the Chinese version of Memorial Delirium Assessment Scale to describe postoperative delirium after hip surgery. Front Aging Neurosci. 2014;6. <https://doi.org/10.3389/fnagi.2014.00297>

10. Slor CJ, Adamis D, Jansen RWMM, Meagher DJ, Witlox J, Houdijk APJ, et al. Validation and psychometric properties of the Delirium Motor Subtype Scale in elderly hip fracture patients (Dutch version). Arch Gerontol Geriatr. 2014;58(1):140-4. <https://doi.org/10.1016/j.archger.2013.07.014>
